# Holstein dairy cows lose body condition score and gain body weight with increasing parity in both pasture-based and total mixed ration herds

**DOI:** 10.3168/jdsc.2022-0246

**Published:** 2022-08-06

**Authors:** I.J. Lean, D.B. Sheedy, S.J. LeBlanc, T. Duffield, J.E.P. Santos, H.M. Golder

**Affiliations:** 1 Scibus, Camden, NSW, Australia 2570; 2 Dairy UP, University of Sydney, Camden, New South Wales, Australia 2570; 3 Department of Population Medicine, Ontario Veterinary College, University of Guelph, Guelph, Ontario, Canada; 4 Department of Animal Sciences, University of Florida, Gainesville 32611

## Abstract

Body condition scoring (BCS) and body weight (BW) are observations associated with labile tissue reserves, health, and reproduction efficiency of dairy cows. The effect of parity (1 through to ≥5) and feeding system (pasture-based and TMR) on BCS and BW were evaluated utilizing raw data sets from 16 retrospective studies that totaled 24,807 Holstein cows across 3 nations (Australia, Canada, and the United States). Linear regression models were used to investigate the 5 outcome variables of precalving BCS, peak milk BCS, change in BCS from precalving to peak milk, and peak milk BW and their respective associations with parity and feeding system. To help control for the influence of calendar time, study treatment protocols when applicable, and genetic change, all outcome variables were center-transformed around each study group mean. Including feeding system as a covariate improved model fit for most outcome variables; however, the relative effect size of parity was generally much greater than feeding system effect size. Parity 2 cows had the lowest precalving BCS of −0.087 [95% confidence interval (CI): −0.107, −0.065] less than the mean, whereas parity 1 cows had the greatest, 0.068 (95% CI: 0.043, 0.092) above mean, regardless of feeding system. Peak milk BCS overall decreased with increasing parity (parity 1 to parity ≥5: −0.13, 95% CI: −0.19, −0.08) and BCS change during the transition period monotonically decreased with increasing parity (parity 1 to parity ≥5: −0.22, 95% CI: −0.26, −0.17). Peak milk BW monotonically increased with increased parity (parity 1 to parity ≥5: 114 kg, 95% CI: 104, 125). A waffle plot was used to present the proportions of cows, by parity, that were partitioned into “low BCS and low BW,” “low BCS and high BW,” “high BCS and low BW,” or “high BCS and high BW” groups. Cows were assigned either a high or low status by being above or below their specific centered study group means, respectively. Considering a null hypothesis of 25% per BCS-BW category, there was a striking change in category from parity 1 cows that were predominantly in the “high BCS and low BW” category (61.2%) to parity ≥5 cows that were predominantly in the “low BCS and high BW” category (55.5%). The study supports studies showing increased weight and change in BCS with increased parity. We highlight the associations among production system, BCS, BW, and parity.

## Highlights

• Body condition score decreases and BW increases in older cows.
• Parity had a greater effect on BCS and BW than feeding system.
• Second-parity cows have the lowest pre-calving BCS.

## Graphical Abstract

**Figure:**
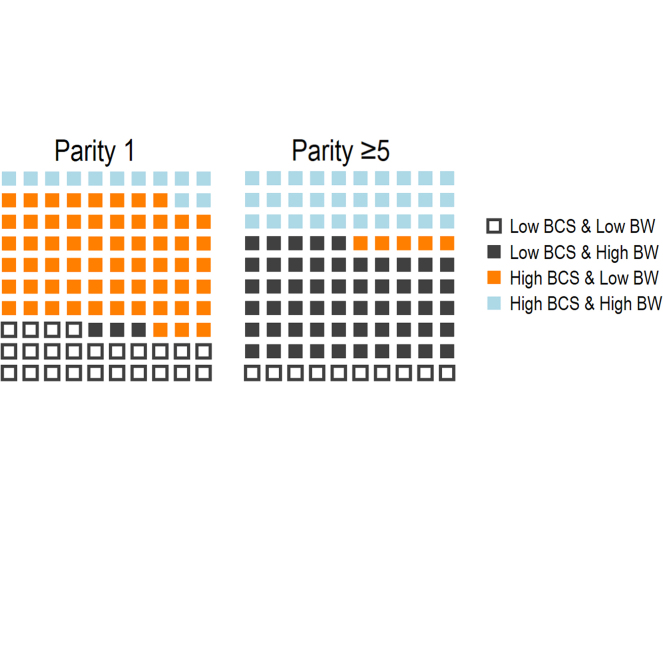
**Summary:** Body condition scoring (BCS) and body weight (BW) measurements are associated with health and reproductive efficiencies of cows. We used raw data sets from 16 studies to evaluate associations of parity and feeding system (pasture-based or total mixed ration) with BCS at precalving and peak milk, and change in BCS, and BW at peak milk. With increasing parity, there is a general decrease in BCS and increase in BW regardless of feeding system, with most young cows having low BW and high BCS and older cows having high BW but low BCS.

Body condition score in dairy cattle is a rapid visual assessment that is highly associated with available energy reserves, primarily mobile fat reserves (Edmondson et al., 1989). Though different rating systems exist, a low score universally reflects low energy stores or emaciation and a high score with excessive energy stores or obesity (Roche et al., 2004). During early lactation, dairy cows are under a strong homeorhetic drive to mobilize energy reserves to produce large quantities of milk (Bauman and Currie, 1980; Baumgard et al., 2017), resulting in a loss of body condition that typically reaches a nadir after the first 40 to 100 DIM (Gallo et al., 1996). Excessive BCS loss during this period is associated with increased disease incidence, reproductive inefficiencies, and potentially poor cow welfare (Roche et al., 2009). Greater parity dairy cows often experience a greater BCS loss compared with nulliparous cows during the early postparturient period and older cows are also associated with an increased risk of removal from the herd due to reproductive failure, disease, and death (Roche et al., 2009; Pinedo et al., 2010).

Body weight also reflects the protein, fat, and energy reserves of cattle (Botts et al., 1979; Gregory et al., 1998; Thorup et al., 2013). There is an imperfect association between BCS and BW that may be influenced by factors such as rumen fill, stage of pregnancy and lactation, and udder fill as well as composition and location of labile tissue pools. Body weight is also associated with parity, with heifers entering the lactation herd typically at around 80% of their mature BW (Berry et al., 2011; Berry and Evans, 2022). Different feeding systems are also reported to influence BCS and BW, with herds from TMR systems typically having higher total BCS and BW than those from pasture-based systems (Washburn et al., 2002; Roche et al., 2007). However, the influence of feeding system on within-herd BCS and BW during the parturition period is less clearly understood and may be similar between systems (Roche et al., 2007). It is important to understand the association of BCS and BW with parity and management systems as this may provide insight into increased risks for disease, reproductive failure, and survival of older cows and how to manage these risks.

In this study we examine associations of parity with precalving and peak milk BCS, changes of BCS between precalving and peak milk observations, and peak milk BW for Holstein cows in different production systems (pasture-based or TMR) across 3 countries using retrospectively collected data. We hypothesized that BCS and BW of dairy cows are associated with cow parity and that BCS and BW are different between the management system types of TMR or pasture-based systems.

The studies used to produce the database for this study were approved by the relevant Animal Care and Use Committees at the time of conduct.

A convenience sample of 16 amalgamated data sets from previous studies were used in this study. In general, commercial dairies were purposively selected for use in the original studies based on good record keeping and a history of performance that suggested capability of maintaining attention to detail congruent with successful trial conduct. The study contributors had conducted large, prospective studies that allowed a rigorous evaluation of the original study hypotheses. Only Holstein or Holstein-Friesian cattle (n = 24,807) were included, as there were few other cow breeds in the database.

Individual study inclusion criteria were being an observational or randomized controlled trial that provided details on parity and either BCS or BW or both. Further details of individual studies that met the criteria are included in Lean et al. (2022a,b). The inclusion criterion for pasture-based study farms was grazing throughout the study period; herds fed partial mixed rations and herds fed concentrates in the parlor were categorized as pasture based. Body condition score data were available from 14 studies, and BW data were available from 8 studies.

The outcome variables investigated were precalving BCS, peak milk BCS and BW, and change in BCS between precalving and peak milk observations. One precalving (−30 to 0 DIM) BCS data point and one peak milk (30 to 110 DIM) BW and BCS data point were entered into the database per cow per study, with only one cow per study. When there were multiple precalving BCS measures per cow, the closest data point to calving was chosen. The peak milk BW and BCS values were determined by the observations obtained at the closest postcalving time point to peak milk, as estimated from herd test or weekly milk yield data. Cows were not required to have a data point for all BW and BCS variables. When an intervention was applied in a herd according to the original study's protocol, treatments within the herd were considered as separate groups for statistical analysis purposes. All outcome variables were center-transformed around the study group mean to control for individual study treatment effects on study outcomes and help mitigate the effects of temporal, genetic, and other clustered, unobserved covariates between each study group.

All statistical analysis was conducted using Stata Version 16 (StataCorp). Initial data evaluation included tabulation of data and visual appraisal of BCS and BW for normality of distribution.

As the database constructed for this study utilized retrospective data, it was possible to compute the statistical power achieved post hoc. Adjustments were made for DIM and feeding system and clustering of study groups, with α set to 0.05. Centered peak milk BCS in heifers was 0.08 (n = 5,165, SD 1.38) and −0.07 for parity ≥5 cows (n = 1,385, SD 0.78). Post-hoc power was calculated as 99.98% (Stata: *power twomeans*). The unit of interest in this study was the cow and statistical significance was set at an α of 0.05.

Ordinary least-squares linear regression and multilevel mixed-effect linear regression (Stata:*regress* and *mixed*, respectively) were used to evaluate the effects of parity and system on BCS, BW, and BCS change between precalving and peak milk. The effects of group were controlled by either assigning group as a random effect in *mixed* or clustered-robust standard errors in *regress*, with the model structure being ultimately determined by minimizing Akaike information criterion (**AIC**). Nesting of group within feeding system was investigated but did not explain group variation beyond a 2-level model. Days prepartum or DIM at time of BCS assignment/BW observation were investigated as covariates, as either continuous, polynomial, categorical, or centered-mean variables. Interactions between system, parity, and time of observation were evaluated. A manual, forward stepwise model was used with model selection determined by minimum AIC. Regression residuals were formally tested for normality with the Shapiro-Francia normality test and heteroskedasticity visually assessed with residual versus fitted value figures.

The Stata code *marginsplot* was utilized to produce graphical summaries of the regression results for cow parity, comparing system where applicable. A waffle plot was used to display the proportions of cows above and below group-centered means for both BCS and BW at peak milk, creating 4 partitions, and each plot was stratified by parity.

The data set mean study size was 1,198 Holstein cows (range: 14–10,958), with a total of 24,807 cows. There were 89.1% (n = 22,112) fed in TMR systems and 10.9% (n = 2,695) pasture based. For the TMR herds, there were 34.7% parity 1 (n = 7,670), 29.9% parity 2 (n = 6,604), 18.8% parity 3 (n = 4,159) 9.4% parity 4 (n = 2,073), and 7.2% parity ≥5 (n = 1,606). In the pasture-based herds, there were 25.6% parity 1 (n = 691), 22.3% parity 2 (n = 600), 21.1% parity 3 (n = 569), 12.7% parity 4 (n = 341), and 18.3% parity ≥5 (n = 494). There were 12.9% (n = 3,198) enrolled cows in AU, 22.5% (n = 5,576) in CAN, and 64.6% (n = 16,033) in the United States. In all cases, the intraclass correlation of study group was best controlled with clustered-robust standard errors in simple linear regression models, according to AIC.

The precalving BCS (−30 to 0 d; mean −7.9, SD 8.1) was evaluated in 12,168 cows across 212 study groups. Parity 1 cows had significantly greater BCS than all other parities, whereas parity 2 cows had significantly less BCS than all other parities, regardless of system of management (Table 1). Parity 3, 4, and ≥5 did not differ significantly from each other (Table 1). The relationship between management precalving BCS and parity for the 2 system types was generally consistent. A notable exception was that pasture-based parity 1 cows had BCS significantly greater than parity >1 pasture-based cows (0.20, 95% CI: 0.11, 0.29) compared with parity 1 cows in TMR systems, which were also significantly greater, but numerically closer to the mean TMR precalving BCS (0.06, 95% CI: 0.04, 0.09; Figure 1). The direct contrast between pasture-based parity 1 and TMR parity 1 was −0.14 (95% CI: −0.24, −0.05). The finding that parity 2 precalving BCS was lower than the centered-mean BCS in both production systems may indicate that neither system is adequately maintaining or recuperating body reserves of parity 1 after postcalving BCS loss nadir. It has been reported that parity 1 cows do not regain lost BCS as quickly as multiparous cows (Berry et al., 2006b; Roche et al., 2007) and that this may be reflected by lower-than-expected BCS of parity 2 cows in our data sets and others (Berry et al., 2011). However, the parity 2 cows in pasture and TMR systems weighed 51.6 kg of BW (95% CI: 41.2, 62.0) greater than parity 1, indicating that BCS and BW are differentially affected by increasing parity.

**Table 1:** Effects of parity and management system on measures of BCS at precalving (−30 to 0 DIM) and peak lactation (30 to 110 DIM), the BCS change between precalving and peak milk observations, and BW (kg) at peak lactation^1^

Model parameter	BCS			BW Peak milk
	Precalving	Peak milk	Change^2^	
Parity (1 referent)				
2	−0.31 ± 0.047 (<0.001)	−0.07 ± 0.020 (0.001)^a^	−0.05 ± 0.017 (0.002)	38.26 ± 8.873 (<0.001)
3	−0.22 ± 0.056 (<0.001)^a^	−0.08 ± 0.026 (0.002)^b^	−0.13 ± 0.021 (<0.001)^a^	67.64 ± 10.283 (<0.001)
4	−0.25 ± 0.048 (<0.001)^a^	−0.09 ± 0.039 (0.030)^ab^	−0.14 ± 0.028 (<0.001)^a^	81.08 ± 12.343 (<0.001)
≥5	−0.22 ± 0.065 (0.001)^a^	−0.10 ± 0.041 (0.012)^a^	−0.22 ± 0.025 (<0.001)	96.37 ± 9.804 (<0.001)
System-parity interaction (pasture referent)				
TMR:parity 1	−0.14 ± 0.048 (0.004)	0.04 ± 0.030 (0.192)	—	−6.26 ± 8.526 (0.468)
TMR:parity 2	0.02 ± 0.013 (0.132)	−0.03 ± 0.016 (0.066)	—	20.50 ± 5.369 (0.001)
TMR:parity 3	0.03 ± 0.022 (0.203)	0.02 ± 0.021 (0.428)	—	32.04 ± 5.585 (<0.001)
TMR:parity 4	0.09 ± 0.026 (0.001)	0.00 ± 0.034 (0.921)	—	26.72 ± 10.081 (0.013)
TMR:parity ≥5	0.00 ± 0.031 (0.929)	−0.02 ± 0.036 (0.561)	—	30.30 ± 6.696 (<0.001)
Days (precalving/in milk)	−0.0037 ± 0.001 (0.007)	0.03 ± 0.012 (0.024)^3^	—	0.21 ± 0.039 (<0.001)
Precalving BCS	—	—	−0.64 ± 0.043 (<0.001)	—
Intercept	0.20 ± 0.046 (<0.001)	0.05 ± 0.019 (0.020)	0.09 ± 0.013 (<0.001)	−53.83 ± 8.303 (<0.001)
Cow count	12,168	15,657	2,803	4,694

a,b Coefficients sharing a letter in the group label are not significantly different at the 5% level.

1 All measure outcome variables and day covariates were centered on their respective study group means. Each cell in the table reports regression coefficients ± SE and *P*-values.

2 Interaction between parity and precalving BCS; parity 2 coefficient = 0.22 ± 0.046 (<0.001); parity 3 coefficient = 0.12 ± 0.057 (0.034); parity 4 coefficient = 0.10 ± 0.073 (0.158); parity ≥5 coefficient = 0.11 ± 0.082 (0.170).

3 Categorical covariate: 31–70 DIM (referent) and 71–110 DIM.

**Figure 1:**
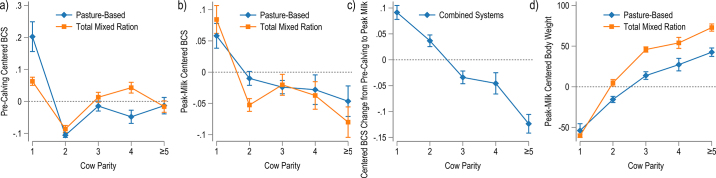
Predictions from linear regression models of (a) precalving centered BCS, (b) peak milk centered BCS, (c) the change in centered BCS from precalving to peak milk, and (d) peak milk centered BW (kg) for different cow parities and management systems are depicted. All outcome variables were center-transformed around study group mean values. Error bars are standard errors.

Body condition score at peak milk was investigated with 15,657 cows in 169 groups. The covariable DIM was dichotomized into 30–70 and 71–110 DIM due to heterogeneity of observation date among studies and to improve model fit according to AIC. There was a consistent decline in BCS with increasing parity, with all parities having significantly lower BCS than the parity 1 heifers and parity ≥5 cows having the lowest body condition of all parities (Table 1). There was a numerically larger decline (*P* = 0.06) in peak milk BCS from parity 1 to parity 2 cows observed in TMR herds (−0.14, 95% CI: −0.20, −0.08) compared with pasture-based herds (−0.09, 95% CI: −0.11, −0.03; Figure 1).

The loss of body condition between precalving and peak milk, with 2,803 cows in 110 study groups, increased monotonically for all parities, with only parity 3 and 4 cows having nonsignificant pairwise comparisons (Table 1). This finding is consistent with previous studies that examined BCS change during the transition period (Roche et al., 2009). Including system or timing of observations did not improve the regression fit. A greater precalving BCS was associated with losing more body condition by peak milk (Table 1). High precalving body condition and excessive loss of BCS during the transition period has been associated with increased incidence of disease, including milk fever and ketosis, and reduced reproductive efficiency (Roche et al., 2009).

The BW at peak lactation (30 and 110 DIM, mean 50.9, SD 16.2) was assessed in 4,694 cows in 32 study groups. Body weight increased monotonically with increasing parity and was significantly different for all pairwise parity comparisons. Despite parity 1 cows not being different with regard to their predicted centered BW across management systems (−6.26 kg, 95% CI: −23.7, 11.12), the interaction of parity and management system shows a greater spread around the centered BW in TMR herds (parity 1 to parity 5: 132 kg, 95% CI: 126, 139) compared with pasture-based herds (parity 1 to parity 5: 96 kg, 95% CI: 76, 116) (Table 1, Figure 1).

The distributions of cows with observations for both BCS and BW at peak lactation were categorized as being above or below the centered group mean for each respective variable and graphically depicted in a waffle plot (Figure 2). Generally, as cows increased in parity they increased in BW but had BCS less than the mean. The null hypothesis is that, by parity, each of the 4 potential BCS-BW categories would contain 25% of cows. There were more than expected parity 1 cows with above mean BCS and below mean BW (61.2%). By contrast, parity ≥5 cows had many more than expected below mean BCS and greater than mean BW (55.5%; Figure 2). Previous studies have focused on individual cows and reported that the correlation between BW and BCS differ with increased parity. As such, the predicted BW change associated with a unit change in BCS differs across parities (parity 1: 44 kg/BCS unit to parity ≥6: 55 kg/BCS unit; Berry et al., 2006a, 2011). However, a consolidated description on a large and diverse population of cows with BCS-BW grouping, stratified by parity, has not previously been presented in a focused manner as reported here. The results were replicated when (1) heifers were removed from the analysis; with concerns they would skew the group mean BW and (2) non-Holstein breeds of cattle were analyzed (data not shown). A study on 50 cows also reported that nulliparous cows had greater BCS and lower BW than multiparous cows throughout the transition period; however, percentage BW loss was independent of parity and was associated with reproduction indices (Sakaguchi, 2009).

**Figure 2:**
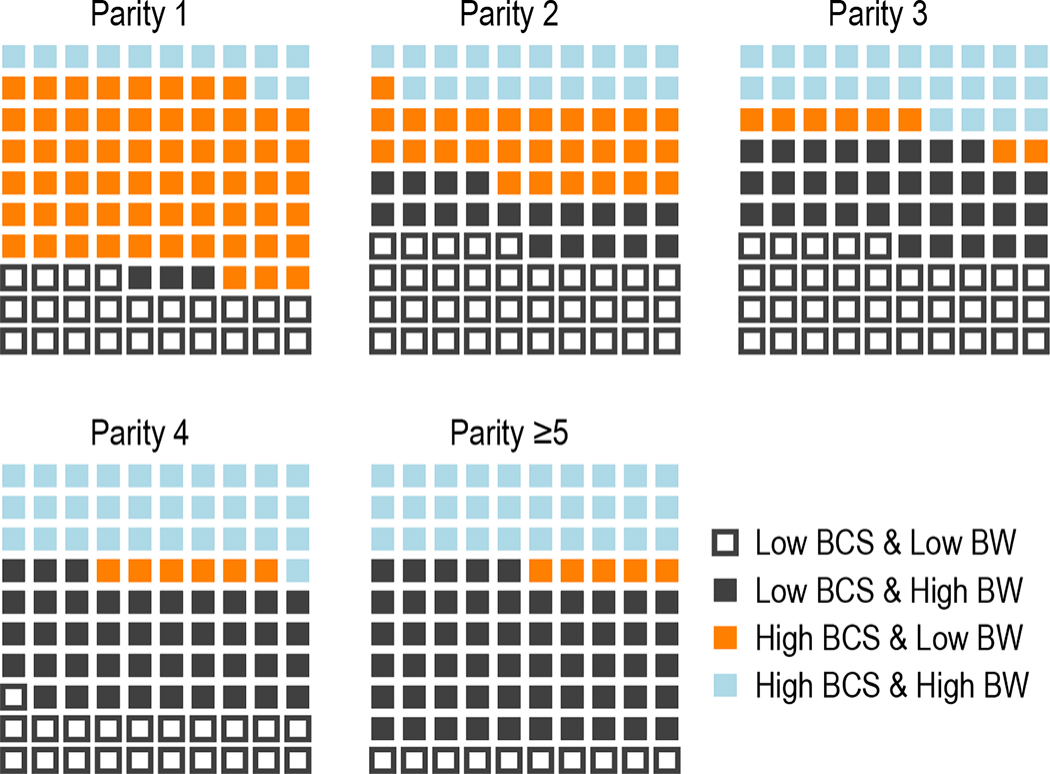
Proportion of cows in each category of BCS (low BCS ≤ group mean, high > group mean) and BW (low BW ≤ group mean, high > group mean) by parity at peak milk.

The finding of increased proportion of BW and lower BCS, with increased parity may have importance in identifying pathways that influence reproduction and health. The labile protein reserves are considered important determinants of health, reproduction, and production (Bell et al., 2000; Ji and Dann, 2013; Lean and DeGaris, 2021). Characterizing the responses to protein nutrition fed in the dry and transition period has been challenging (Husnain and Santos, 2019); however, nulliparous cows benefited more from increased MP before calving than multiparous cows and cows producing greater than 36 kg/d also increased production with increased MP before calving. Rodney et al. (2016) suggest that cows that can adjust to anabolic stimuli, such as additional protein supply, increase yield of milk protein, maintain a higher milk protein percentage, and improve reproductive performance. Yan et al. (2009) analyzed body composition in 146 head of Holstein-Friesian cattle and evaluated some parity effects. Live weight and empty-BW increased with parity; however, there was little difference in ratio of empty-BW to live weight for parity 1 (0.72) vs. parity ≥3 (0.73); the lipid, CP, and ash content (kg) numerically increased with parity. None of those comparisons were statistically significant possibly because the standard deviations were high, especially for lipid (Yan et al., 2009). Given the increased BW with parity, it is unclear what implications the pattern of change in BCS and BW with parity has for labile tissue pools that could influence immune and inflammatory response and provide support for lactation and health.

Limitations to this study include using single time point observations for BCS and BW. It is known that the nadir of body condition loss during early lactation is different across parities (Roche et al., 2009) and is not a simple linear relationship; however, by including DIM as a covariate, we help control for observation time. It was not possible to compare inter- and intraobserver variability for BCS assignment, though all assignments were performed by skilled practitioners with years of cattle experience. We attempted to control for genetic influence on BCS/BW outcome variables by using group-centered means, which requires an assumption that intragroup variation associated with genetics was similar across study groups.

Body condition loss during the transition period is strongly associated with cow parity, with older cows losing a much greater amount of labile energy reserves than younger cattle. Parity 2 cows had lower BCS results than expected at both precalving and peak milk observations. The use of waffle plots to visualize BCS-BW is unique and easily highlights the dramatic change in the body as cows age.
